# A rat Satb1 truncation causes neurodevelopmental abnormalities recapitulating the symptoms of patients with *SATB1* mutations

**DOI:** 10.1038/s41401-025-01588-6

**Published:** 2025-06-26

**Authors:** Zhi-bin Hu, Wei-tang Liu, Yi-wei Li, Ling Hu, Ying Huang, Xi-yue Liu, Qiong Zhang, Yu-bing Wang, Jia-yin Chen, Ze-xuan Li, Si-xin Tu, Li Zhao, Ning-ning Song, Oded Klavir, Yu-qiang Ding

**Affiliations:** 1 State Key Laboratory of Brain Function and Disorders, MOE Frontiers Center for Brain Science, Institutes of Brain Science, Fudan University, Shanghai, 200032 China; 2 Institute of Infectious Disease and Biology, Fudan University, Shanghai, 200032 China; 3 Laboratory Animal Center, Fudan University, Shanghai, 200032 China; 4 Key Laboratory of Arrhythmias, Ministry of Education, East Hospital, and Department of Anatomy and Neurobiology, Tongji University School of Medicine, Shanghai, 200092 China; 5 School of Psychological Sciences, The University of Haifa, Haifa, 3103301 Israel; 6 The Integrated Brain and Behavior Research Center (IBBRC), University of Haifa, Haifa, 3103301 Israel; 7 Huashan Institute of Medicine (HS-IOM), Huashan Hospital, Fudan University, Shanghai, 200040 China

**Keywords:** neurodevelopmental disorders, SATB1 syndrome, microcephaly, intellectual disability

## Abstract

The special AT-rich sequence binding protein 1 (SATB1) has been linked to neurodevelopmental disorders (NDDs) including developmental delay, intellectual disabilities (ID) and autism spectrum disorder (ASD). But the underlying biological mechanisms are still not fully understood. In this study we generated a rat model with a truncated Satb1 protein. We showed that *Satb1* mutant caused growth retardation, microcephaly, altered ultrasonic vocalization and delayed neurobehavioral development in mutant pups as well as social and cognitive behavior deficits in adult mutants, mimicking the typical clinical characteristics of *SATB1*-associated NDDs. Injection of a GABAergic enhancer clonazepam (0.04 mg/kg, i.p.) effectively alleviated the abnormal social and cognitive behaviors in *Satb1* mutant rats. Finally, RNA sequencing analysis further revealed a potential role of Satb1 in a cortical transcriptional regulatory network associated with NDDs including ID and ASD. Our results confirm the crucial roles of *SATB1* in the pathogenesis of NDDs and provide insights into treatment strategies for *SATB1*-associated NDDs.

## Introduction

Neurodevelopmental disorders (NDDs), including intellectual disability (ID), autism spectrum disorder (ASD), attention deficit/hyperactivity disorder, and communication disorders, may result in severe delays or abnormalities in growth, alterations in brain architecture, and limited cognitive functions and adaptive skills [1–3]. Genome-wide association studies have identified a certain number of genes associated with NDDs, some of which belong to the category of transcription factors/chromatin remodelers [1, 4–6]. Mutations in transcriptional regulators such as *MECP2*, *ASH1L*, *POGZ*, and *ARID1B* lead to different severe phenotypes in humans and related rodent models [7–10]. Recently, human genetic studies have shown that copy number variants and de novo mutations in *SATB1*, another transcription factor, are strongly associated with ID and ASD [11–13]. Clinical case reports have revealed that almost all individuals with *SATB1* mutations exhibit neurodevelopmental delay, most suffer from ID, and some are diagnosed with behavioral disturbances, including ASD-related behaviors [13–15]. In addition, the clinical characteristics associated with *SATB1* variants include microcephaly, epilepsy, endocrine/metabolic abnormalities, and facial dysmorphisms. However, the neurobiological functions of *SATB1*, particularly the pathogenesis of NDDs induced by its dysfunction, remain unclear.

The Satb1 protein specifically binds to DNA sequences via its AT-rich DNA-binding domain [16], and previous studies have focused mainly on its role in tumorigenesis and the immune system [17–20]. Satb1 is expressed in the developing brain [21] and is required for the development of interneurons derived from the medial ganglionic eminence [22, 23]. Moreover, Satb1 regulates the expression of multiple immediate early genes and is implicated in the regulation of neuronal plasticity in the mouse brain [24]. Satb1 affects the dendritic morphology of retinal ganglion cells by regulating the expression of Contactin 5 [25]. Recently, SATB1 has attracted attention as a risk gene for Parkinson’s disease [26, 27], and studies have examined its effects on the development and survival of midbrain dopaminergic neurons [28, 29]. These prior studies have established several mouse models with *Satb1* deletion, but the short lifespan precludes an exploration of the contributions of *SATB1* dysfunction to the pathogenesis of NDDs in these mouse models [24, 30]. Additionally, behavioral observations from *Satb1* conditional knockout mice or local inhibition of *Satb1* expression via virus injection fail to mimic the symptoms of patients with *SATB1* mutations [22, 29, 31]. Therefore, no comprehensive studies have defined the role of *SATB1* in NDDs.

In this study, we generated the first rat model expressing a truncated Satb1 protein to investigate the consequences of *Satb1* mutation in vivo, with a focus on neurological aspects. Fortunately, *Satb1* mutant rats are viable and can grow into adulthood. *Satb1* mutant rats exhibit growth retardation, as indicated by reductions in body weight and body size, and delayed eye opening. Microcephaly observed in patients is also present in *Satb1* mutant rats. Behavioral analyses of these rats revealed neurodevelopmental delay, cognitive inflexibility, and deficient social behaviors. Dendritic morphology and neuronal activity are also disrupted in *Satb1* mutant rats, possibly through differentially expressed genes in the transcriptome. A decreased number of SST-positive interneurons leads to impaired GABAergic system function in *Satb1* mutant rats. Furthermore, treatment with a GABAA receptor agonist partially rescues cognitive dysfunction and social behaviors in *Satb1* mutant rats. Taken together, these observations provide the first in vivo evidence and mechanistic insights into the roles of *SATB1* dysfunction in the pathogenesis of NDDs.

## Materials and methods

### Generation of *Satb1* mutant rats

The transcription activator-like effector nuclease (TALEN)-encoding plasmid targeting the *Satb1* gene was obtained from Shanghai Taiting Biotechnology Co., Ltd. (Shanghai, China). The target sequence for the Satb1 TALEN was located within exon 1 of the *Satb1* gene (NM_001012129). The TALEN mRNA was synthesized from plasmids that were linearized via *Not*I digestion and subsequently purified [32]. The procedures for the collection of fertilized oocytes from Sprague–Dawley (SD) rats, microinjection, and embryo transfer were then performed as previously described [33].

### Genotyping and maintenance of the rats

For genotyping of *Satb1* mutant rats, genomic DNA was extracted from rat tail tips. PCR was performed using standard techniques. The forward primer (5′- acc ttg gtg cag att cc -3′) and reverse primer (5′- acc ttg gtg cag att cc -3′) were used to determine the genotype of each rat. The PCR products (size: 680 bp) were sent for Sanger sequencing with the forward primer or treated with the *Ban*II restriction endonuclease. Depending on the sequence length, the expected sizes of the homozygous mutant and wild-type digestion products were 680 bp and 315 + 365 bp, respectively.

*Satb1* heterozygous SD rats were crossed with WT SD rats for at least 6 generations, followed by heterozygous × heterozygous mating to generate offspring for all the experiments in this study. The rats were housed by sex (2–4 rats per cage) in a room at a constant temperature of 23 °C with a 12 h light/dark cycle and provided *ad libitum* access to food and water. All experiments involving animals were reviewed and approved by the Animal Committee of the Department of Laboratory Science, Fudan University, Shanghai, China.

### Nissl staining, immunohistochemistry, BrdU labeling and in situ hybridization

Embryos at different stages and rats at different postnatal ages were perfused with warm saline followed by 4% paraformaldehyde in 0.1 M phosphate-buffered saline (PBS). After cryoprotection with 30% sucrose in PBS, the brains were embedded in O.C.T. compound, sectioned into 25-μm-thick slices, and then mounted onto glass slides. For Nissl staining, slides were defatted for 30 min in xylene, followed by rehydration through a series of solutions consisting of 100% ethanol, 95% ethanol, and deionized water, each for 5 min. Slides were then stained with 0.3% cresyl violet for 5 min and rinsed with two changes of deionized water. The sections were subsequently dehydrated for 5 min in 95% ethanol, 100% ethanol, and xylene and sealed with neutral gum.

For immunohistochemistry, the following primary antibodies were used: mouse anti-Satb1 (1:200; SC376096, Santa Cruz), rabbit anti-Cux1 (1:1000; OB-PRB038-01, Oasis Biofarm), mouse anti-Tle4 (1:100; SC365406, Santa Cruz), rabbit anti-Pax6 (1:500; PR-278P-100, Covance), guinea pig anti-Tbr2 (1:500; OB-PGP022-01, Oasis Biofarm), and rabbit anti-PH3 (1:300; 06-570, Upstate). The sections were incubated with primary antibodies at 4 °C overnight, followed by an incubation with either biotin-conjugated secondary antibodies (Vector Laboratories) or secondary antibodies conjugated to Alexa fluorochromes (Molecular Probes, Invitrogen) for 2 h and finally with Cy3-conjugated streptavidin (1:1000; 016160084, Jackson ImmunoResearch) and Hoechst 33258 (1:2000; 94403, Sigma Aldrich) for 1 h. For BrdU labeling, pregnant rats received one pulse of BrdU (60 mg/kg body weight, B9285, Sigma) and were euthanized 1 h later. The brain slices were sequentially subjected to treatment with sodium citrate (0.01 M, pH 6.0) at 95 °C for 10 min, HCl (2 N) at 37 °C for 20 min, and sodium borate (0.1 M, pH 8.5) at room temperature for 10 min. The slices were then immunostained with a rat anti-BrdU antibody (1:1000; OBT0030G, Accurate), as described above. In situ hybridization was performed as described previously [32]. The following RNA probes were used: Satb1, Gad67, PV, and SST.

### Western blots

Brain tissues were dissected at different developmental stages and homogenized in M-PER reagent using a protease inhibitor cocktail (GRF101, EpiZyme). After centrifugation, the supernatants were collected, and loading buffer (LT101, EpiZyme) was added. For each sample, protein was separated via SDS‒PAGE and transferred to a nitrocellulose membrane. The membrane was subsequently blocked with TBST containing 5% skim milk powder for 1 h at room temperature (RT). After blocking, the membrane was incubated with primary antibodies. The following primary antibodies were used: mouse anti-Satb1 (1:200; SC376096, Santa Cruz) and mouse anti-GAPDH (1:2000; LF206, EpiZyme) as an internal control. After the primary antibody incubation, the membranes were incubated with an HRP-conjugated goat anti-mouse (IgG) secondary antibody (1:1000; LF101, EpiZyme) or an HRP-conjugated goat anti-rabbit (IgG) secondary antibody (1:2000; LF102, EpiZyme) and exposed for chemiluminescent detection. The protein levels were analyzed using ImageJ and normalized to the level of GAPDH.

### RNA-Seq and quantitative real-time PCR (qRT-PCR)

The RNA-seq assay was performed by Shanghai Biotechnology Corporation. Briefly, poly(A) RNA was purified from total RNA and then converted to double-stranded cDNA; the resulting cDNA samples were sequenced using standard Solexa protocols. The sequencing reads were mapped to the rat genome using TopHat (version 1.0.13). Avadis NGS (version 1.3) was used to calculate the reads per kilobase per million mapped reads values. The criteria for identifying differentially expressed genes (DEGs) in the RNA-seq analysis were a fold change >1.2 and a *P*-value < 0.05. The analysis of DEGs was performed using “Metascape” (https://metascape.org/).

For real-time qRT-PCR, brain tissues were dissected at different developmental stages, and RNA was extracted with RNAiso Plus (9108Q, TaKaRa Biotechnology) and then converted to cDNA with the PrimeScript™ RT reagent kit (RR047A, TaKaRa Biotechnology). qRT-PCR was performed using TB Green premix (RR820A, TaKaRa Biotechnology) on a QuantStudio 6 Flex Real-Time PCR System ABI-Q7 (Applied Biosystems). The primers used are listed in the Supplementary file: Table S1. All expression data were normalized to GAPDH transcript levels. The values obtained from the mutant rats were then normalized to those of the control rats.

### Golgi staining and dendritic spine analysis

Golgi staining and the spine analysis were performed as previously described [34], with minor modifications. A standard commercial kit (PK401, FD Rapid Golgi Stain Kit, FD NeuroTechnologies, Inc.) was used for Golgi staining of rat brain sections. Images of 150-μm-thick coronal cryosections were captured, and the Z-stack images were merged using a microscope (DS-Ri2, Nikon). Images of at least twelve neurons from layers II/III of the somatosensory cortex of each rat brain were captured to quantify the dendritic length and spine density. The spine density of secondary or tertiary dendritic branches was determined. Spine morphologies are classified as mushroom, stubby, or thin spines based on the relative sizes of the spine head and neck.

### Behavioral tests

All behavioral tests were conducted randomly using age-matched mutant rats and littermate control rats during the light phase of the circadian cycle, specifically between 9:00 a.m. and 4:00 p.m. Male and female rats were used in a balanced manner in this study. Prior to the initial experimental session, all adult rats were handled for ~15 min daily over a minimum of 7 days. On the test day, all the rats were allowed a 30 min habituation period following transfer to the behavioral test environment. For drug treatment, we performed the experiments 30 min after the intraperitoneal injection of 0.04 mg/kg clonazepam into the rats. The experimenter remained blinded to the genotypes of the subjects throughout the testing and analysis procedures.

#### Ultrasonic vocalization test

Rat pups emit ultrasonic vocalizations (USVs) at frequencies ranging from 30 to 65 kHz when isolated from their dam. These vocalizations are interpreted as a form of communication. Each pup separated from its mother was placed in a plastic container, wherein USVs were recorded for a duration of 300 s. A subsequent analysis of USVs was performed utilizing the automatic selection mode of the Sonotrack system, with data adhering to established criteria being included in the statistical analyses, as outlined in previous studies [35].

#### Surface righting test

The surface righting reflex measures the motor ability of rat pups to be able to flip onto their feet from a supine position. Pups were placed on their backs on a flat plastic plate and held in position for 5 s. Then, the pups were released, and the time it took the pup to return to the prone position was recorded. A total of 60 s was allowed for each trial, and the results of three trials were averaged for analysis.

#### Cliff avoidance test

The cliff avoidance test reflects strength and coordination in rodent pups. Each pup was placed on the edge of the acrylic board to ensure that its forepaws, digits, and snout were the only parts that were affected by age; then, the pup was released, and the time it took the pup to withdraw its paws and snout was recorded. A total of 30 s was allowed for each trial, and the results of three trials were averaged for analysis.

#### Negative geotaxis test

The negative geotaxis test reflects motor coordination in rodent pups. Each pup was placed facing down a 45° slope, held for 5 s, and then released, and the time when the pup turned facing upward was recorded. Each experiment lasted for up to 120 s, and the results of three trials were averaged for analysis.

#### Grip strength test

The grip strength test reflects the strength of all four paws at the same time. Each pup was placed on a wire mesh screen and allowed to adjust for 5 s. Then, the screen was inverted slowly to 180°, and the angle when the pup fell off was recorded. The results of three trials were averaged for analysis.

#### Suspension tests

Suspension tests were conducted to evaluate the forelimb and hindlimb strength of the rat pups. Each pup was allowed to grasp a wire strung across a pencil cup with felt padding with both forepaws. The latency to release from the wire was recorded across three separate trials that were then averaged for analysis. The pup was placed facing downward into a 100 mL conical tube with the hindlimbs hanging over the edge to determine the hindlimb suspension ability. The latency to release from the edge of the conical tube was recorded across three separate trials that were then averaged for analysis.

#### Five-trial social memory test

Social recognition memory was quantified through a five-trial habituation-dishabituation paradigm in which the subject rats engaged in freely accessible reciprocal interactions with novel versus familiar conspecifics. Briefly, the test rats were individually housed overnight before the behavioral assay. On the test day, the rat remained in its home cage and was presented with an age- and sex-matched rat for 4 successive 1 min trials, interspersed with 10 min intervals. In the final trial, another novel rat was introduced. The close interaction time was scored.

#### Open field test

Locomotor ambulation was measured to assess activity, exploration, and anxiety-like levels in the open field test. Specifically, the rats were placed in the center of a translucent acrylic apparatus measuring 50 cm × 50 cm (L × W) and allowed to freely explore the arena for 15 min. Movement was quantified using EthoVision XT 15 software (Noldus). The ambient light intensity was adjusted to 100 lux. The total distance traveled in the whole arena and the time spent in the central arena were recorded.

#### Y maze test

The Y maze spontaneous alternation test was used to assess repetitive behavior and working memory. The rats were placed at the end of one arm of a symmetric Y maze with arms 50 cm long, 10 cm wide, and 35 cm high and allowed to explore freely for 10 min. The number and order of entries into each of the three arms were analyzed. A correct spontaneous alternation occurred when the rat entered a different arm in each of the three consecutive arm entries. The percentage of alternations was calculated as follows: total correct spontaneous alternations/(total arm entries – 2) × 100.

#### Novel object recognition test

The novel object recognition test is a two-trial cognitive paradigm that has been used to assess recognition memory. Before training, the test rat was habituated individually to the arena (40 cm × 40 cm) for 10 min for three consecutive days. During the training phase, the rat was placed in the arena in the presence of two identical objects that they could explore for 5 min. For the test, one of the objects was replaced by a new one with a distinguishable shape. The time spent in close proximity was recorded, and the recognition index was calculated as the time spent exploring the novel object/(time spent exploring the novel object + time spent exploring the familiar object).

#### Three-chamber test

The three-chamber social approach task was used to test sociability and social novelty preference. The apparatus consisted of an acrylic box (90 cm × 48 cm, each chamber being 30 cm × 48 cm) without a lid, and the box was partitioned into three chambers with 10-cm openings between the chambers. The task consisted of three consecutive 10-min trials. In trial 1, the test rat was placed in the center of the three-chamber unit, where two empty wire cages were located in the left and right chambers, and the rat was allowed to freely explore to habituate to the whole apparatus. Trials 2 and 3 tested sociability and social novelty preference, respectively. In trial 2, an unfamiliar, sex- and age-matched rat (S1) was placed in one cage, and the other was placed with an object (O). During trial 3, a novel rat (S2) was introduced to replace the object, and the rat from trial 2 remained in the same cage to serve as the familiar stimulus. During each trial interval, the rat was placed back into its home cage. The time spent in close proximity to the rat (S1, S2) or object (O) was analyzed.

#### Morris maze test

The Morris water maze was used to measure spatial memory and learning in rodents. The maze was a circular tank with a 160-cm diameter and water at a depth of 100 cm, maintained at 22 °C ± 1 °C, as described previously [32, 36], with slight modifications. The maze was divided into four equal quadrants by designating two orthogonal axes, the ends of which demarcated four cardinal points: north (N), south (S), east (E), and west (W). The traces of the rats were recorded by a video camera hung above the center of the pool. During the training period, the rat was trained to find the hidden platform over 6 consecutive days, with four trials per day. The escape platform (15 cm in diameter) was positioned in the middle of quadrant II (target quadrant), approximately 5 cm below the surface of the water. The rat was gently released into the water from different starting points and allowed to locate the hidden platform within 60 s. If the rat failed to find the platform within 60 s, it was manually guided to the platform. For the probe test on the 7th day, 24 h after the last training session, the platform was removed, and each rat was allowed 60 s to search for the platform in the pool, with the starting location opposite to the previous position. The movement of each rat was monitored using EthoVision XT 15 software (Noldus). The escape latency to find the platform, total distance traveled, average velocity, total distance to the platform, and duration in the platform zone were automatically analyzed by the software.

### In vivo electrophysiology

The rats were anesthetized with isoflurane (RWD) and fixed in a stereotaxic apparatus. Sixteen-channel electrodes were implanted in the somatosensory cortex of the rats (coordinates: 4 mm anterior from the bregma, 0.6 mm lateral from the midline, and 1.02 mm below the pia). On the 15th day, local field potentials (LFPs) from the somatosensory cortex of freely moving rats were recorded (APPOLO II, Yige Biological Company). The data were analyzed with MATLAB (Mathworks, MA). The pWelch formula was used to estimate the power spectral density (PSD) of the 200 s artifact free fragments of each sample. The segment length of the Hamming window was 5000 data points, and the overlap was 2500 data points. The power spectrum was integrated into the following frequency bands for analysis: δ (0.5–4 Hz), θ (4–8 Hz), α (8–13 Hz), β (13–30 Hz), and γ (30–100 Hz).

### Statistical analysis

Statistical analyses were performed using GraphPad Prism 8 software. All values are presented as the means ± SEMs. The number of samples indicates biological replicates and is indicated with scattered dots in the figures. Comparisons were performed using Student’s *t* test, multiple *t* test, or two-way ANOVA with the Bonferroni correction, as mentioned in each figure legend. The results were considered significant when the *P* value was <0.05.

## Results

### Satb1 expression in the developing and adult brain

**Fig. 1:**
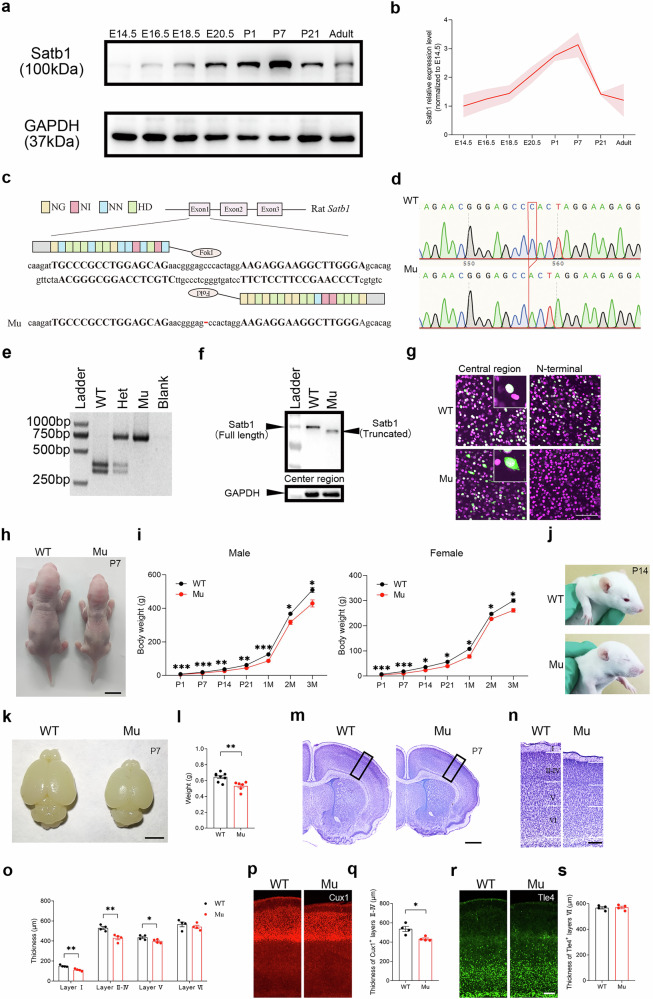
A Satb1 frameshift mutation results in a truncated protein, leading to growth retardation and cortical developmental defects in mutant rats. **a**, **b** Temporal pattern of the Satb1 protein expression levels in the rat cortex. **c** Schematic of the *Satb1* TALEN and its recognition sequence in the ATG area of exon 1 of Satb1. **d** Sanger sequencing data around the TALEN spacer area of wild-type (WT) and *Satb1* mutant rats. **e** A representative image showing the genotyping results obtained after *Ban*II digestion. The DNA fragment lengths of the size markers are shown on the left. Het, heterozygote; Mu, mutant. **f** Western blot data showing that the truncated Satb1 protein is detected by the Satb1 antibody against the central region in mutant rats. **g** Visualization of the truncated Satb1 protein by the Satb1 antibody against the central region but not the one against the N-terminus in mutant rats. Notably, the Sab1 protein (green) is localized within the nuclei of WT cortical neurons, whereas the truncated Satb1 protein is also present in the cytoplasm of the mutants. The nucleus is shown with Hoechst (magenta). **h**, **i** Compared with that of WT control rats, the body weight of mutant rats was lower. Scale bar, 1 cm. *n* = 8–11 rats in each group. Two-way ANOVA with the Bonferroni correction was used. *P < 0.05, **P < 0.01, and ***P < 0.001. **j** Delayed eye opening in *Satb1* mutant rats at postnatal day (P) 14. **k** Representative images showing a reduction in the brain size of *Satb1* mutant rats compared with WT control rats at P7. Scale bar, 5 mm. **l** Decreased brain weight in *Satb1* mutant rats. *n* = 6–8 rats in each group. Student’s *t* test. **P < 0.01. **m** Nissl-stained coronal sections showing a reduced size of the mutant brain compared with the WT control brain. Scale bar, 1 mm. **n**, **o** Quantification of the thickness of the cortical layers shown by Nissl staining. Reductions are evident in layers I-V but not in layer VI in mutant rats compared with WT control rats. Scale bar, 200 mm. *n* = 4 rats in each group. Multiple *t* tests. *P < 0.05 and **P < 0.01. **p**, **q** Cux1 immunostaining revealed a reduced thickness of layers II-IV, but the thickness of layer VI was not changed, as shown by Tle4 immunostaining (**r**, **s**), in mutant rats compared with WT control rats. Scale bar, 200 μm. *n* = 4 rats in each group. Student’s *t* test. *P < 0.05.

We first examined the Satb1 expression profile in the developing cerebral cortex of rats. Western blots showed that Satb1 protein levels increased during the embryonic and early postnatal stages, peaked at P7, and were sustained at lower levels into adulthood (Fig. 1a, b). Next, we investigated the Satb1 distribution by immunohistochemistry and in situ hybridization. Although immunostaining detected no positive signals at E14.5, in situ hybridization revealed that the *Satb1* mRNA was expressed throughout the cortex, including the neurogenic ventricular zone (VZ) and subventricular zone (SVZ) at this stage (Supplementary file: Fig. S1a, b), when the rat cortex begins to develop. Moreover, the expression patterns of Satb1 protein and mRNA in the cortex were very similar to each other after E18.5 (Supplementary file: Fig. S1a, b). In the postnatal brain, Satb1 expression was distributed throughout the cortex and persistent into adulthood. The dynamic expression of Satb1 in the cerebral cortex suggests that Satb1 is likely involved in cortical neurogenesis and in the regulation of brain functions.

### Generation of *Satb1* mutant rats

We employed a rat model to investigate whether Satb1 dysfunction plays a critical role in the pathogenesis of neurodevelopmental disorders (NDDs). We used TALEN-mediated gene editing of Sprague‒Dawley oocytes to generate rats harboring a mutant sequence in *Satb1* exon 1, which is upstream of most identified human mutations [13], as shown in Fig. 1c. F0 mutation carrier lines were obtained after the microinjection of TALENs into fertilized eggs. Sanger sequencing verified a frameshift mutation in exon 1 of *Satb1* due to a 1-bp deletion (Fig. 1d), and the mutation site could also be distinguished by *Ban*II cleavage (Fig. 1e). Given the presence of multiple ATG initiation codons in the 5’ region of *Satb1*, we hypothesized that the Satb1 protein likely undergoes translational reinitiation from an in-frame ATG codon downstream of the mutation site in mutant rats. We performed Western blot analyses using two antibodies targeting distinct epitopes to validate this hypothesis. The SATB1 (Santa Cruz, sc-376096) antibody, which recognizes an epitope in the central region of SATB1 that is predicted to be preserved within the truncation, detected the full-length protein in WT rats and the truncated form in mutant rats (Fig. 1f). Consistently, immunostaining of brain sections with a central region-specific antibody confirmed the presence of the truncated Satb1 protein in the mutant rats (Fig. 1g). Notably, the truncated Satb1 protein displayed an abnormal cytoplasmic localization in the mutant cortex (insert, Fig. 1g). As expected, the N-terminal-specific antibody showed similar distributions of Satb1-positive neurons in the WT cortex relative to those revealed by the central region-specific antibodies, but no signals were observed in the mutant cortex (Fig. 1g). These results collectively indicate that *Satb1* mutant rats express an N-terminally truncated Satb1 protein.

### *Satb1* mutant rats exhibit growth retardation and microcephaly

Unlike the *Satb1* knockout mouse models [24, 30], our mutant rats were viable and could survive into adulthood, enabling an exploration of the pathogenesis of *SATB1*-associated NDDs. Compared with WT littermate controls, *Satb1* mutant rats had a smaller body size, reduced body weight (two-way ANOVA; *P* < 0.0001; Fig. 1h, i) and delayed eye opening (Fig. 1j). In addition, as microcephaly has been reported in some *SATB1* mutant carriers [13] and *Satb1* is widely expressed in the developing cerebral cortex, we speculated that *Satb1* mutation may affect cortical development. First, the brain size and weight were obviously reduced in the mutants (Student’s *t* test; *P* = 0.0029; Fig. 1k, l). The cellular architecture of the cortex was examined by Nissl staining. Although the layered organization in the cortex was grossly normal, the thickness of layers I-V was significantly reduced, with no change in layer VI in the mutants compared with the WT controls (Student’s *t* test; *P* = 0.0024; Fig. 1m–o). This finding was further confirmed by Cux1 (a marker for layers II-IV) and Tle4 (a marker for layer VI) immunostaining (Student’s *t* test; *P* = 0.0118; Fig. 1p‒s).

### Reduced proliferation of neural stem cells in *Satb1* mutant embryos

The presence of microcephaly in *Satb1* mutant rats prompted us to investigate whether this microcephaly was caused by defective neurogenesis during the embryonic stages. We first examined the number of progenitors in the ventricular zone (VZ) and subventricular zone (SVZ) at embryonic day (E) 16.5, a stage of active neurogenesis. Immunostaining for Pax6 and Tbr2, two transcription factors that determine the commitment of apical/basal progenitors in the VZ and SVZ [37], respectively, revealed no significant differences between mutant rats and WT controls (Supplementary file: Fig. S2a, b, e, f). Next, immunostaining for phosphorylated histone 3 (PH3), a specific marker for proliferating progenitors in the late G2 and M phases (Supplementary file: Fig. S2i, j), also revealed no difference in the VZ between the two genotypes. In addition, proliferating cells at S phase were labeled with bromodeoxyuridine (BrdU) [38], and the number of BrdU-positive cells in the VZ and SVZ was comparable between the two genotypes 1 h after pulse labeling (Supplementary file: Fig. S2m, n). These results suggest that the proliferation of neural stem cells remains unaffected in *Satb1* mutant rats at E16.5.

In contrast, significant reductions in the numbers of PH3-positive (Student’s *t* test; *P* = 0.0018; Supplementary file: Fig. S2k, l) and BrdU-labeled stem cells (Student’s *t* test; *P* = 0.0105; Supplementary file: Fig. S2o, p) were observed in the VZ of *Satb1* mutant rats at E18.5, particularly in the basal region, compared with WT controls, indicating diminished proliferation of neural progenitors at this stage, although the populations of Pax6- and Tbr2-positive cells in the VZ and SVZ did not differ significantly between *Satb1* mutant rats and WT controls at this stage (Supplementary file: Fig. S2c, d, g, h). Collectively, these results indicate that Satb1 is involved in the proliferation of neural stem cells/progenitors in the neurogenic region and that reduced proliferation may contribute to the microcephaly of *Satb1* mutant rats.

### Altered ultrasonic vocalizations (USVs) and delayed neurobehavioral development in *Satb1* mutant pups

**Fig. 2:**
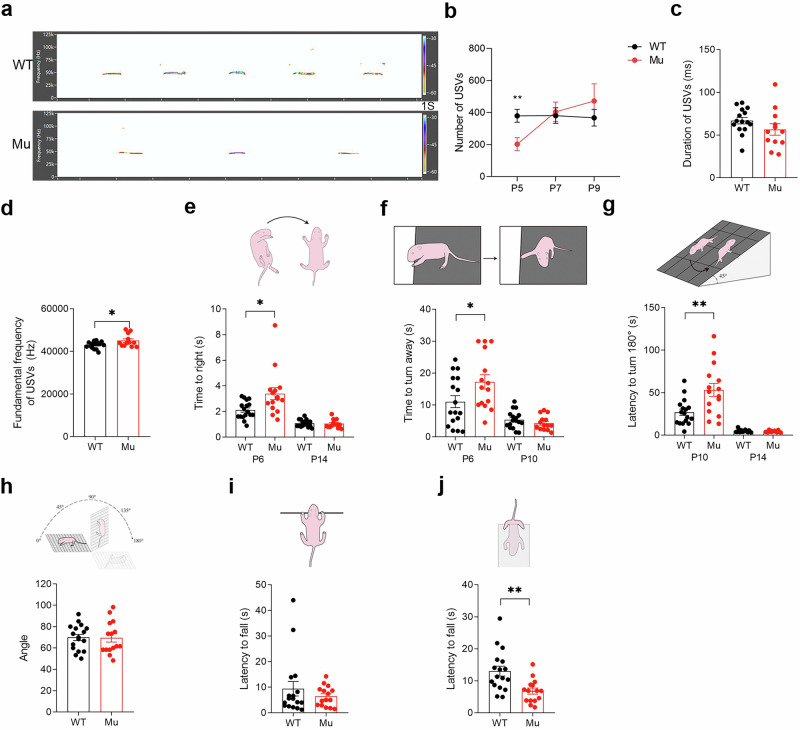
Impaired communication ability and delayed neurobehavioral development in *Satb1* mutant pups. **a** Representative recordings of USVs from WT and *Satb1* mutant rats at P5. **b** The quantification of the USV rate revealed a decrease in the total number of USVs in *Satb1* mutant pups compared with WT controls at P5. **c** No significant differences in the mean duration of USVs were detected between WT and *Satb1* mutant rats at P5. **d** A higher fundamental frequency of USVs is present in *Satb1* mutant rats than in WT controls at P5. *n* = 12–15 rats in each group. **e** Righting reflex. Compared with WT control rats, *Satb1* mutant rats exhibited a longer latency to the righting reflex at P6. **f** Cliff avoidance reflex. Compared with WT control rats, *Satb1* mutant rats exhibited a longer latency to the cliff avoidance reflex at P6. **g** Negative geotaxis reflexes. Compared with WT control rats, *Satb1* mutant rats exhibited longer latency to negative geotaxis reflexes at P10. **h** Grip strength test. *Satb1* mutant rats exhibited normal claw strength in the grip strength test. **i** Forelimb suspension test. *Satb1* mutant rats presented normal forelimb strength. **j** Hindlimb suspension test. *Satb1* mutant rats exhibited reduced hindlimb strength. *n* = 13–17 rats in each group. Multiple *t* test for (**b**), Student’s *t* test for others. *P < 0.05 and **P < 0.01.

In addition to growth retardation, speech and motor delay/abnormalities are common in infants and children with *SATB1* mutations [13]; therefore, we conducted a comprehensive behavioral characterization of mutant pups. We first examined USVs of postnatal day (P) 5–9 pups, which are essential for mother–infant social interaction, and isolation-induced USVs are commonly used to evaluate communication ability [39]. The number of USVs decreased significantly in the mutant pups at P5 but was comparable to that in the WT controls at P7 and P9 (Student’s *t* test; *P* = 0.0051; Fig. 2a–c). However, the fundamental frequency of the USVs increased significantly in the mutants (Student’s *t* test; *P* = 0.0296; Fig. 2d). Thus, *Satb1* mutant pups presented deficits in communication behavior, which may reflect some aspects of the language deficit in the patients who carry *SATB1* mutations.

Neonatal rodents develop rapidly during the first 2 postnatal weeks, and we next conducted several tasks (i.e., reflexes and strength) to assess a possible motor delay in mutant pups at P6, P10, and P14 [40]. The mutants exhibited increased latencies in the test of righting reflexes (Student’s *t* test; *P* = 0.0112; Fig. 2e), cliff avoidance (Student’s *t* test; *P* = 0.0401; Fig. 2f), and negative geotaxis reflexes (Student’s *t* test; *P* = 0.0031; Fig. 2g) at P6, but these abnormalities were no longer apparent at P10 or P14. Although the mutants exhibited normal claw strength in the grip strength assessments (Fig. 2h) and maintained typical forelimb strength during the forelimb suspension tests (Fig. 2i), impaired hindlimb strength was observed, indicating the presence of hypotonia (Student’s *t* test; *P* = 0.0023; Fig. 2j). Taken together, *Satb1* mutant pups presented physical manifestations of gross motor delay and hypotonia, which are present in individuals with *SATB1* mutations.

### Impaired cognitive ability of *Satb1* mutant rats

**Fig. 3:**
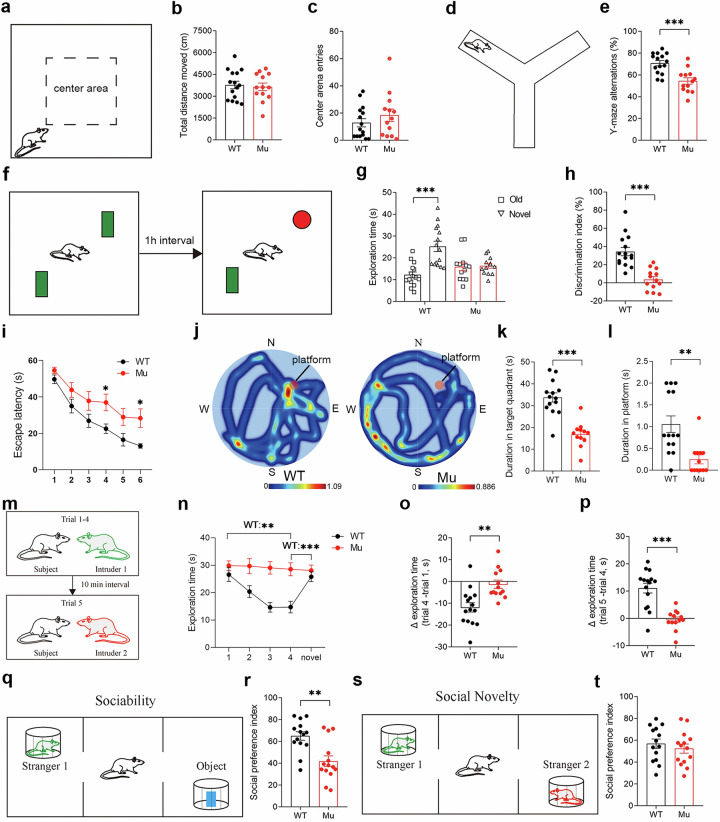
Impaired cognitive and social behaviors in adult *Satb1* mutant rats. **a**–**c** Open-field test. No significant differences were observed in the total distance traveled or number of center area entries. **d**, **e** Y-maze test. Compared with those in WT control rats, spontaneous alternation rates were lower in *Satb1* mutant rats. **f**–**h** Novel object recognition test. WT control rats spent more time exploring the novel object, whereas *Satb1* mutant rats spent comparable amounts of time exploring the two objects. This difference is also evidenced by the comparison of the discrimination indices. **i**–**l** Morris water maze test. Escape latency (**i**). *Satb1* mutant rats spent more time finding the platform during the training phase (**j**), exhibited a shorter duration in the target quadrant (**k**), and a shorter duration in the platform location in the probe phase (**l**) than WT rats. **m**–**p** Five-trial social memory test. The total time WT rats spent exploring the same intruder in trials 1–4 gradually decreased but increased in trial 5 (presence of a novel intruder), whereas *Satb1* mutant rats spent similar interaction times in all 5 trials (**n**). Differences in exploration time between trials 4 and 1 (**o**) and between trials 5 and 4 (**p**) were not significantly different in *Satb1* mutant rats, whereas a substantial reduction in the former and an increase in the latter were observed in WT control rats. **q**, **r** Three-chamber social tests for assessing sociability. WT controls showed a greater preference index for S1 in the presence of a subject, which was not detected in *Satb1* mutant rats. **s**, **t** Three-chamber social tests for social novelty. No significant differences were observed in the preference index for novel S2. *n* = 12–15 rats in each group. Two-way ANOVA with the Bonferroni correction was used for (**i**), and Student’s *t* test was used for the other variables. *P < 0.05, **P < 0.01, and ***P < 0.001.

Given that patients with *SABT1* mutations exhibit ID and other behavioral abnormalities [13], we performed a battery of behavioral tests to examine the cognitive and emotional behaviors of adult mutant rats. We first exposed the rats to the open field test to measure spontaneous locomotor activity. No significant differences in the distance traveled or number of center entries were observed in the mutant rats compared with WT control rats (Fig. 3a–c). The Y-maze task was used to examine working memory, and the mutants presented a significantly lower spontaneous alternation rate than the WT controls (Student’s *t* test; *P* = 0.0002; Fig. 3d, e). We also assessed recognition memory using the novel object recognition test. The WT rats spent more time approaching the novel object (Student’s *t* test; *P* < 0.0001; Fig. 3f, g), whereas the mutants did not show any preference for exploring either familiar or novel objects (Student’s *t* test; *P* < 0.0001; Fig. 3h). In addition, spatial memory was assessed by the Morris water maze test. During the 6-day acquisition phase, the mutants spent more time finding the platform on the 4th and 6th days (two-way ANOVA; *P* = 0.0349; Fig. 3i), indicating that the capacity for spatial learning was limited in the mutants. In the spatial probe trial performed on Day 7, the mutants spent less time in the target quadrant (Student’s *t* test; *P* < 0.0001; Fig. 3j, k) and the platform quadrant (Student’s *t* test; *P* = 0.0014; Fig. 3l) than the WT rats did, indicating that spatial memory was impaired. Notably, the total swimming distance and swimming speed were comparable between the two genotypes. Taken together, these results indicate that working memory, cognitive functions, spatial learning, and memory are impaired in *Satb1* mutant rats.

### Deficient social behaviors in *Satb1* mutant rats

*SATB1* was reported to be an ASD-associated transcription factor in recent whole-exome sequencing studies [11, 12, 41], and autistic behaviors have been observed in some patients with *SATB1* mutations [13]. In support of these results, we found that USVs were impaired in mutant pups, as described above (Fig. 2a–d). We thus conducted multiple behavioral tests to investigate whether other ASD-like behaviors were present in adult mutant rats. Social recognition abilities were evaluated via a 5-trial test [42]. The WT controls spent gradually decreasing amounts of time interacting with the familiar intruder rat during trials 1 to 4, but the time spent interacting with a novel rat increased to baseline levels in trial 5 (Student’s *t* test; *P* < 0.0001; Fig. 3n). In contrast, the mutants maintained equal interaction times in all 5 trials, indicating a defective social recognition ability (Fig. 3m–p). We also performed a 3-chamber social test to further assess social behavior [34]. In the sociability test, mutants spent significantly less time interacting with stranger 1 (S1) rats than WT controls did, and the decreased preference index also suggested the presence of defective sociability in mutants (Student’s *t* test; *P* = 0.001; Fig. 3q, r). On the other hand, the mutants preferred to explore stranger 2 (S2) rats, similar to WT controls, which was indicative of normal levels of social novelty in the mutants (Fig. 3s, t). These results indicate that *Satb1* mutant rats exhibit deficient social behaviors.

### Reduced dendritic complexity and spine density of cortical neurons in *Satb1* mutant rats

**Fig. 4:**
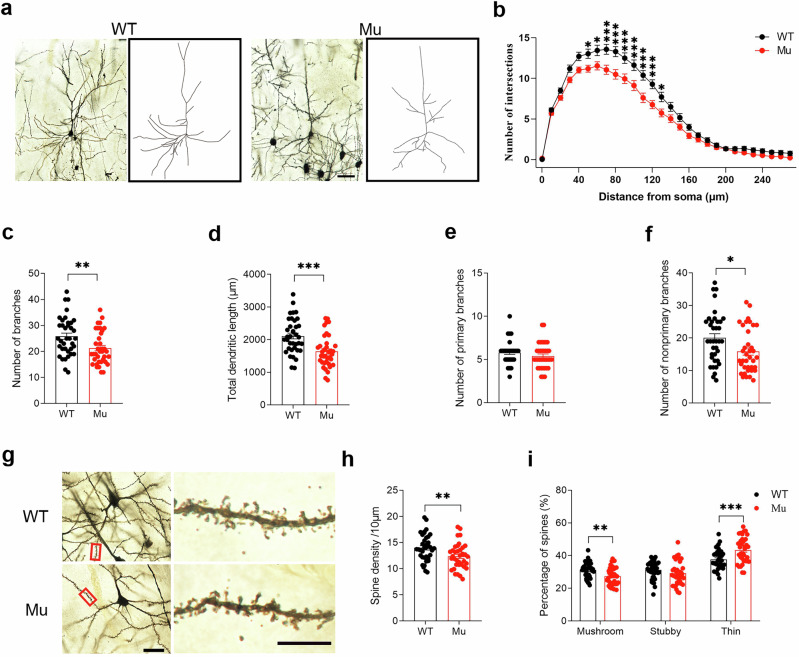
Reduced dendritic complexity and spine density of cortical neurons in *Satb1* mutant rats. **a** Representative images of Golgi-stained pyramidal neurons in layers 2/3 of the somatosensory cortex of WT and *Satb1* mutant rats. Scale bar, 50 µm. **b** Sholl analysis showing the reduced dendritic complexity of pyramidal neurons in *Satb1* mutant rats. **c** The number of dendritic branches was lower in *Satb1* mutant rats than in WT control rats. **d** The total dendritic length was shorter in *Satb1* mutant rats than in WT control rats. **e**, **f** No significant differences were observed in the number of primary branches, but the number of nonprimary branches was lower in *Satb1* mutant rats than in WT control rats. **g**–**i** Representative images of dendritic spines in layers 2/3 of the somatosensory cortex (**g**). The spine density (**h**) and proportion of mushroom spines were lower but the proportion of thin spines was higher in *Satb1* mutant rats than in WT controls (**i**). Scale bars (**g**), left panel, 30 µm; right panel, 100 µm. *n* = 37 neurons from 3 rats in each group. Two-way ANOVA with the Bonferroni correction for (**b**), multiple *t* test for (**i**), and Student’s *t* test for the other variables. *P < 0.05, **P < 0.01, and ***P < 0.001.

Having observed abnormal behaviors in mutant rats, we explored the underlying cellular mechanism. To this end, we analyzed the morphology of cortical neurons using Golgi staining. A Sholl analysis was performed to assess the complexity of dendrites in layer 2/3 pyramidal neurons of the somatosensory cortex, and the results showed that the numbers of intersections (two-way ANOVA; *P* = 0.045; Fig. 4a, b) and total dendritic length (Student’s *t* test; *P* = 0.0002; Fig. 4c) were decreased in adult mutant rats (Fig. 4a–f), indicating that the dendritic arborization of cortical neurons was less complex in *Satb1* mutants. Furthermore, we performed a parametric analysis of spines, and the fraction of spines with mushroom, stubby, or thin morphologies was quantified to examine the morphological changes in more detail. The mutant rats presented a significantly decreased spine density (Student’s *t* test; *P* = 0.006; Fig. 4g, h) and altered proportions of mushroom and thin spines. In particular, the proportion of thin spines was increased, but that of mushroom spine was reduced in the mutants compared with the WT controls (Student’s *t* test; *P* = 0.0017; Fig. 4i). These results suggest that the *Satb1* mutation leads to a reduction in the dendritic complexity and spine density of cortical neurons.

### Increased gamma activity in *Satb1* mutant rats

**Fig. 5:**
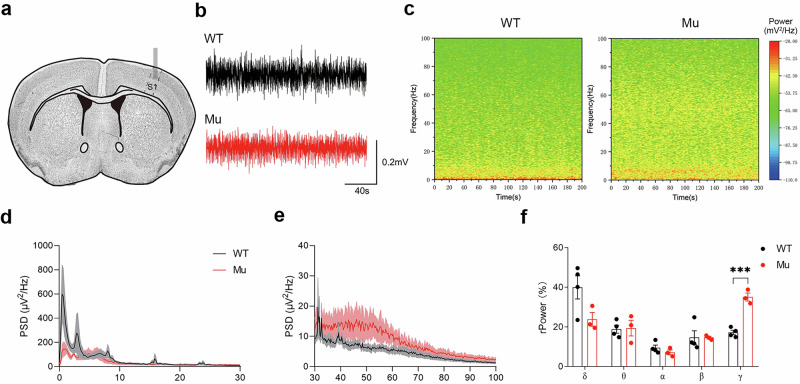
Abnormal EEG in *Satb1* mutant rats. **a**–**c** Electrodes were implanted in the somatosensory cortex, and representative LFPs and spectrograms recorded in the cortex are shown. **d**, **e** EEG power density profile ranging from less than 30 Hz (**d**) to between 30 Hz and 100 Hz (**e**). **f** Histogram showing the relative power in different frequency bands of LFPs in the cortex. Compared with WT control rats, *Satb1* mutant rats exhibit increased gamma power. *n* = 3–4 rats in each group. Multiple *t* test. ***P < 0.001.

Electroencephalography (EEG) recording is a powerful tool for detecting brain dysfunction in individuals with neurodevelopmental and neuropsychiatric disorders [43–45]. Given that approximately 80% of patients with *SATB1* mutations exhibit EEG abnormalities [13], EEG recordings were also performed in our rat model. We implanted electrodes into the somatosensory cortex and recorded local field potentials (LFPs; Fig. 5a–c). In *Satb1* mutant rats, the relative power density in the delta band tended to decrease, but the gamma power increased significantly (Student’s *t* test; *P* = 0.00044; Fig. 5d–f). Increased gamma power is associated with social communication and cognitive impairments in individuals with NDDs [46, 47]; therefore, the disrupted gamma activity may contribute to the abnormal behaviors of mutant rats.

### The activation of GABA signaling rescues abnormal behaviors in *Satb1* mutant rats

**Fig. 6:**
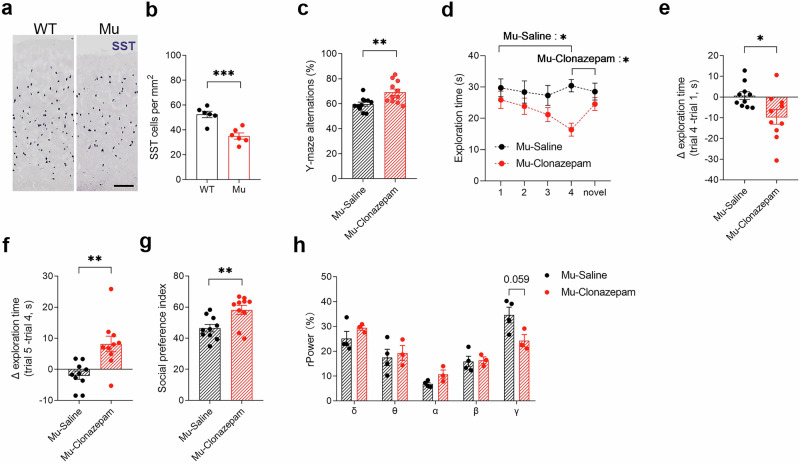
The abnormal behaviors and EEG signals in *Satb1* mutant rats are rescued by clonazepam. **a**, **b** The number of SST-positive interneurons was reduced in *Satb1* mutant rats, as shown by in situ hybridization. *n* = 6 rats in each group. Student’s *t* test. ***P < 0.001. **c** Alternation in the Y-maze test. Treatment with clonazepam rescues working memory deficits in *Satb1* mutant rats. **d**–**f** The time spent exploring the intruder rat in each of the 5 trials in the 5-trial social test. After clonazepam treatment, mutant rats display a gradual reduction in exploration time from trial 1–4 with a familiar intruder and increased exploration time in trial 5 with a novel intruder. Note that the differences in exploration time (s) between trials 4 and 1 (**e**) and between trials 5 and 4 (**f**) were substantially decreased in the former and increased in the latter in *Satb1* mutant rats after clonazepam treatment. **g** The comparison of the preference indices in the 3-chamber social test revealed that clonazepam treatment restored the preference for stranger 2 in *Satb1* mutant rats. *n* = 10–11 rats in each group. Student’s *t* test. *P < 0.05 and **P < 0.01. **h** Compared with saline treatment, clonazepam treatment decreased the increase in gamma power in *Satb1* mutant rats, although the difference was not significant. *n* = 3–4 rats in each group. Multiple *t* test.

Previous studies have reported that Satb1 is required for the development of cortical interneurons in mice [21–23, 25, 31]; thus, we investigated the distribution of interneurons in the cortex to explore whether it has a similar function in rats. Although the numbers of Gad67- and parvalbumin (PV)-positive interneurons were comparable between the mutants and WT controls (Supplementary file: Fig. S3a–d), a significant reduction in the number of somatostatin (SST)-positive interneurons was observed in the cortex of the mutant rats (Student’s *t* test; *P* = 0.00068; Fig. 6a, b). Therefore, we assessed whether the pharmacological modulation of GABA activity could rescue the abnormal behaviors observed in *Satb1* mutant rats, in which the same volume of saline was used as a control. Clonazepam is a benzodiazepine tranquilizer that functions as an agonist for GABAA receptors, and low doses of clonazepam are able to mitigate abnormal behaviors in animal models of neurodevelopmental diseases [10, 48–50]. Treatment with a low dose of clonazepam increased the rate of correct alternation in the Y maze of *Satb1* mutant rats (Student’s *t* test; *P* = 0.0061; Fig. 6c). In the 5-trial social test, clonazepam treatment decreased the time mutant rats spent interacting with the same repeatedly exposed intruder rat in the first 4 trials (Student’s *t* test; *P* = 0.012; Fig. 6d) and significantly increased the time mutant rats spent sniffing the novel rat in the last trial (Fig. 6d–f). In addition, deficient sociability was no longer present in the mutants after clonazepam treatment in the 3-chamber social test (Student’s *t* test; *P* = 0.0061; Fig. 6g), and the increased gamma power was reduced, although it did not reach a significant difference compared with that in the mutants treated with saline (Student’s *t* test; *P* = 0.059; Fig. 6h). However, in the Morris water maze test, clonazepam administration failed to improve the performance of *Satb1* mutant rats throughout both the acquisition phase and probe trial phase (Supplementary file: Fig. S3e-g). These findings suggest that clonazepam is very likely to be a potential pharmacological intervention for *SATB1*-associated NDDs.

### Mutation of *Satb1* alters NDDs and the expression of synaptic-related genes in rats

**Fig. 7:**
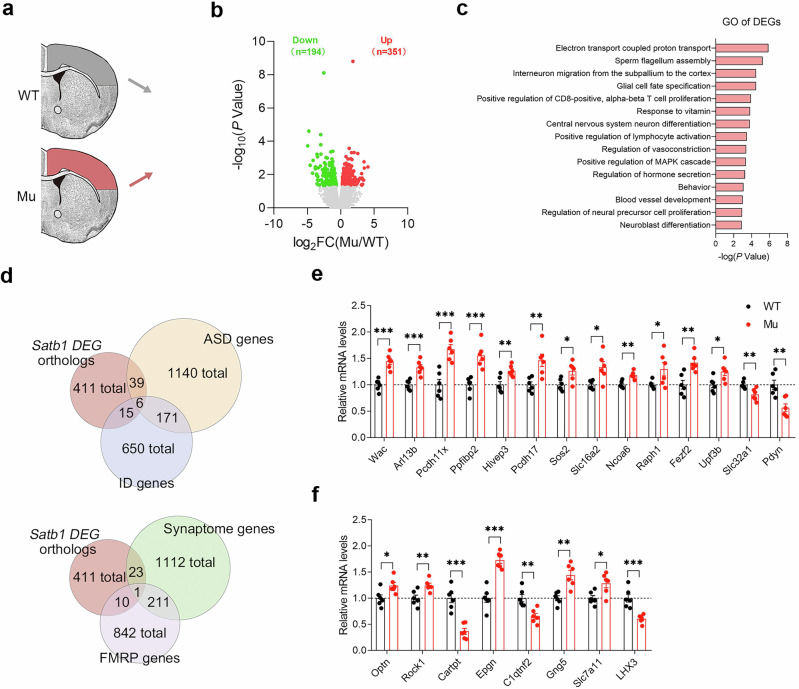
Altered transcriptional profile in the cortex of *Satb1* mutant rats. **a** Scheme of the RNA-seq experiment. **b** Volcano plot of *Satb1* mutant rats vs. WT control rats. **c** Gene Ontology analysis of differentially expressed genes between *Satb1* mutant rats and WT control rats. **d** Venn diagrams identifying the overlap between DEGs and NDD/synaptosome gene databases. **e**, **f** Verification of the alterations in the genes involved in NDDs/synaptosomes and neuron-related pathways. *n* = 3 rats in each group. Student’s *t* test. *P < 0.05, **P < 0.01, and ***P < 0.001.

Given that Satb1 acts as a transcription regulator to govern the expression of downstream genes, we performed RNA sequencing (RNA-seq) to examine changes in the transcriptional network in the cortex of mutant rats. Compared with WT control rats, 545 differentially expressed genes (DEGs) were identified in mutant rats, among which 351 genes were upregulated and 194 genes were downregulated (Fig. 7a, b; Supplementary file: Table S2). Gene Ontology (GO) analyses of the DEGs affected by *Satb1* mutation revealed that the DEGs were enriched mostly in biological processes involving electron transport coupled with proton transport, interneuron migration from the subpallium to the cortex, and central nervous system neuron differentiation (Fig. 7c; Supplementary file: Table S3). In addition, both the positive regulation of the MAPK cascade and the regulation of cell precursor proliferation, which are closely related to the development of the nervous system [51, 52], were also enriched. These results further show that Satb1 plays an important role in neuronal development.

Numerous studies have indicated that shared molecular pathways and changes in synaptic function may explain the various clinical signs associated with NDDs [1, 53, 54]. We compared the DEGs that have a nonambiguous human ortholog with the gene databases for ID [55], ASD [56], FMRP [57], and synaptosomes [58] to further determine the link between *Satb1* and NDDs. As shown in the Venn diagrams, among the 411 human orthologs of DEGs, 21 (5.1%) were associated with ID, 45 (10.9%) with ASD, 11 (2.7%) with FMRP, and 24 (5.8%) with the synaptosome (Fig. 7d; Supplementary file: Table S4). Next, we performed qPCR to validate our RNA-seq data. We confirmed that the expression of genes overlapping with ID (*Arl13b*, *Slc16a2*, and *Upf3b*), ASD (*Wac*, *Pcdh11x*, *Sos2*, *Fezf2*, *Hivep3*, and *Upf3b*), FMRP (*Ncoa6* and *Hivep3*) and synaptosome (*Ppfibp2*, *Pcdh17*, *Raph1*, *Slc32a1*, and *Pdyn*) was altered (Fig. 7e, f). We also confirmed that the expression of genes involved in interneuron migration from the subpallium to the cortex (*Arl13b*, *Pdyn*, *Fezf2*, and *Slc7a11*), regulation of cell precursor proliferation (*Slc16a2*, *Optn*, and *Gng5*), central nervous system neuron differentiation (*Fezf2* and *Lhx3*) and positive regulation of the MAPK cascade (*Rock1*, *Cartpt*, *Epgn*, and *C1qtnf2*) was altered. Taken together, our molecular analyses of mutant rats indicate that *SATB1* is essential for neuronal development and that defects in SATB1 are very likely to be associated with NDDs, including ID and ASD.

## Discussion

NDDs are early-onset mental illnesses, and many patients are diagnosed in early childhood. In the present study, we generated the first *Satb1* mutant rat model and showed that mutation of *Satb1* indeed led to NDD-related phenotypes in rats. We confirmed that the frameshift mutation results in a truncation in the Satb1 protein. The truncated Satb1 protein leads to various physical and behavioral abnormalities, many of which resemble symptoms observed in patients, including microcephaly, postnatal developmental delay, ID, hypotonia, deficient cognitive and social abilities, and altered EEG. Clinical data and the present results strongly support the idea that *SATB1* is a risk gene involved in the pathogenesis of NDDs and that its mutation leads to severe birth defects.

Satb1 is a transcription factor that has been extensively studied for its roles in tumorigenesis and immune cell differentiation. Although previous studies have reported that Satb1 is essential for regulating the maturation of interneurons, it has not been linked to NDDs because existing animal models have a short lifespan [23, 24], and the conditional knockout of *Satb1* in interneurons replicates only some symptoms in patients [22, 31]. Thus, the effects of genetic defects in *Satb1* on behavior and brain development remain obscure. In this study, we used rats as the experimental subjects to investigate the pathogenesis of *SATB1*-associated NDDs. As expected, the *Satb1* mutant rats survived into adulthood, enabling the exploration of possible behavioral changes.

Neurodevelopmental delay is the clinical phenotype of almost all patients with *SATB1* mutations, followed by ID, EEG abnormalities, and various behavioral disturbances, including autistic behaviors such as social impairment and communication abnormalities [13, 14]. USV recordings are routinely assessed in models of NDDs for anxiety- and communication-related phenotypes [59]. *Satb1* mutant rats presented a decrease in the number of USV emissions and an increase in the frequency of USVs, indicating a potential disruption in communication abilities that parallels features observed in patients with ASD [60]. By investigating several developmental reflexes, we observed that *Satb1* mutant rats exhibit a generalized delay in neurobehavioral development during the early postnatal period. In addition to prolonged eye opening, *Satb1* mutant rats presented an extended response time for several developmental reflexes, including surface righting, cliff aversion, and negative geotaxis reflexes. These reflexes require trunk control and dynamic postural adjustments and depend on the intricate integration and communication between sensory input and motor output, which are further influenced by the vestibular system and cerebellar functions [40, 61]. Additionally, findings from fore- and hindlimb suspension tests indicate deficits in hindlimb strength, which may be attributed to hypotonia, a characteristic frequently observed in individuals with *SATB1* mutations.

Furthermore, the results of the behavioral tests of *Satb1* mutant rats in adulthood paralleled the behavioral characteristics of individuals with ID and ASD. Notably, these mutant rats exhibited deficits in working memory, recognition memory, and spatial learning and memory. Additionally, social behavior tests revealed impaired social abilities in mutant rats. Encouragingly, the cognitive and social deficits observed in *Satb1* mutant rats could be restored through treatment with the GABAergic enhancer clonazepam, although this remediation does not directly stem from alterations in synaptic morphological plasticity. Moreover, the modulation of gamma power is crucial for the maintenance of cognitive abilities and social interactions [62, 63]. EEG recordings indicated that low-dose clonazepam administration tended to diminish the increased gamma power in mutant rats, reinforcing our behavioral findings and suggesting that EEG could serve as a reliable biomarker for the diagnosis of neurodevelopmental disorders [44]. These results provide potential therapeutic strategies for the clinical treatment of *SATB1*-related NDDs.

Previous studies have revealed that shared cellular pathways, including aberrant dendritic outgrowth and disruptions in neuronal connectivity, underlie the heterogeneous clinical manifestations characteristic of NDDs [1]. Our analysis of neuronal morphology with Golgi staining revealed that the dendritic branches of pyramidal neurons are simplified in *Satb1* mutant rats. Additionally, notable reductions in the density of dendritic spines and the proportion of mature spines were observed. Furthermore, we recorded LFPs from the sensory cortex of the rats via EEG. We observed that *Satb1* mutant rats presented increased gamma power, which aligns with findings reported for some patients with NDDs. Increased gamma power is associated with communication abnormalities and cognitive deficits [45, 64, 65], which is highly consistent with our behavioral results. Therefore, the behavioral defects of mutant rats can be at least partially attributed to the physiological dysfunction of their brains. Furthermore, abnormalities in interneurons in the brain also lead to increased gamma power [45, 66], and a reduction in the number of SST-positive interneurons was observed in mutant rats. Consequently, while the specific alterations in neuronal subtypes contributing to EEG abnormalities remain ambiguous, increased gamma power is indicative of an excitatory/inhibitory imbalance within the brains of mutant rats. *Satb1* conditional knockout models are needed to address this question in the future.

Clinically reported *SATB1* mutations in humans predominantly manifest through loss-of-function (LOF) mechanisms, frequently involving truncations or missense variants that compromise nuclear localization or the DNA-binding capacity. Although the frameshift mutation in our rat model differs structurally from known human variants, the resulting N-terminal truncation recapitulates critical molecular hallmarks of human LOF mutations. Mechanistically, this truncation eliminates the evolutionarily conserved nuclear localization signal (NLS) within the N-terminus of Satb1, leading to cytoplasmic mislocalization, which may consequently abrogate transcriptional regulatory activity. Notably, Western blot quantification revealed markedly reduced expression levels of the truncated Satb1 protein in mutant rats, which was likely attributable to structural instability and proteasomal degradation, thereby reinforcing the LOF mechanism.

Our RNA-seq analysis revealed that DEGs are involved in neuronal proliferation, differentiation, and migration processes, which are intricately linked to brain development [67]. Although these results are derived from adult samples, at which time the brain structure is fully developed, the regulation of transcription factors has a lasting and far-reaching effect, which provides evidence to explain the microcephaly of mutant rats. Among the identified DEGs, two candidate genes belonging to the protocadherin (PCDH) protein family have been shown to be involved in neuronal development. Notably, the overexpression of *Pcdh11x* in murine cortical neurons leads to a reduction in dendritic complexity, whereas the overexpression of *Pcdh17* results in a decreased density of dendritic spines. These findings are corroborated by the observed increased expression of these genes in mutant rats, which aligns with the diminished dendritic complexity and spine density of pyramidal neurons noted in *Satb1* mutant rats. Although our study did not investigate the direct regulatory effects of Satb1 on DEGs through a ChIP-seq analysis, this aspect was not the primary focus of our current research. In contrast, we propose that the impaired nuclear localization of the Satb1 protein may disrupt its regulatory effects on downstream target genes, potentially leading to aberrant neural development. Furthermore, the truncated form of Satb1 in the nucleus may influence the transcriptional activity of these genes through a gain-of-function mechanism, thereby exerting distinct regulatory effects. Nevertheless, our bulk RNA-seq data reflect the overall genetic landscape of the cortex without delineating region-specific or cell type-specific differences. Consequently, single-cell RNA-seq is warranted for a more nuanced understanding.

In conclusion, we identified a new link between mutations in the *Satb1* gene and the clinical characteristics of individuals with *SATB1*-associated NDDs. Mutation of the *Satb1* gene in rats induced various behavioral deficits that are similar to those observed in people with neurodevelopmental delays, intellectual disabilities, and ASD. RNA-seq confirmed that many of the DEGs are associated with NDDs and synaptosome function, which helps to explain the morphological abnormalities in the pyramidal neurons of *Satb1* mutant rats, such as simplified dendritic branching and reduced maturation of dendritic spines. Additionally, treatment with low-dose clonazepam was shown to alleviate abnormal behaviors and EEG irregularities in adult *Satb1* mutant rats. These findings support the idea that *SATB1* is a candidate for the genetic etiology of *SATB1*-associated NDDs and provide a potential therapeutic approach for patients with SATB1 mutations.

## Supplementary information

Supplementary file: Table S1

Supplementary file: Table S2

Supplementary file: Table S3

Supplementary file: Table S4

Supplementary file: Fig. S1

Supplementary file: Fig. S2

Supplementary file: Fig. S3

Supplementary file Tables and Figure Legends
