# Supplementary figures and images for: A rat Satb1 truncation causes neurodevelopmental abnormalities recapitulating the symptoms of patients with SATB1 mutations
Source: Acta Pharmacol Sin. 2025 Jun 26;46(12):3163–76. doi: 10.1038/s41401-025-01588-6 (PMC12644705; doi:10.1038/s41401-025-01588-6)

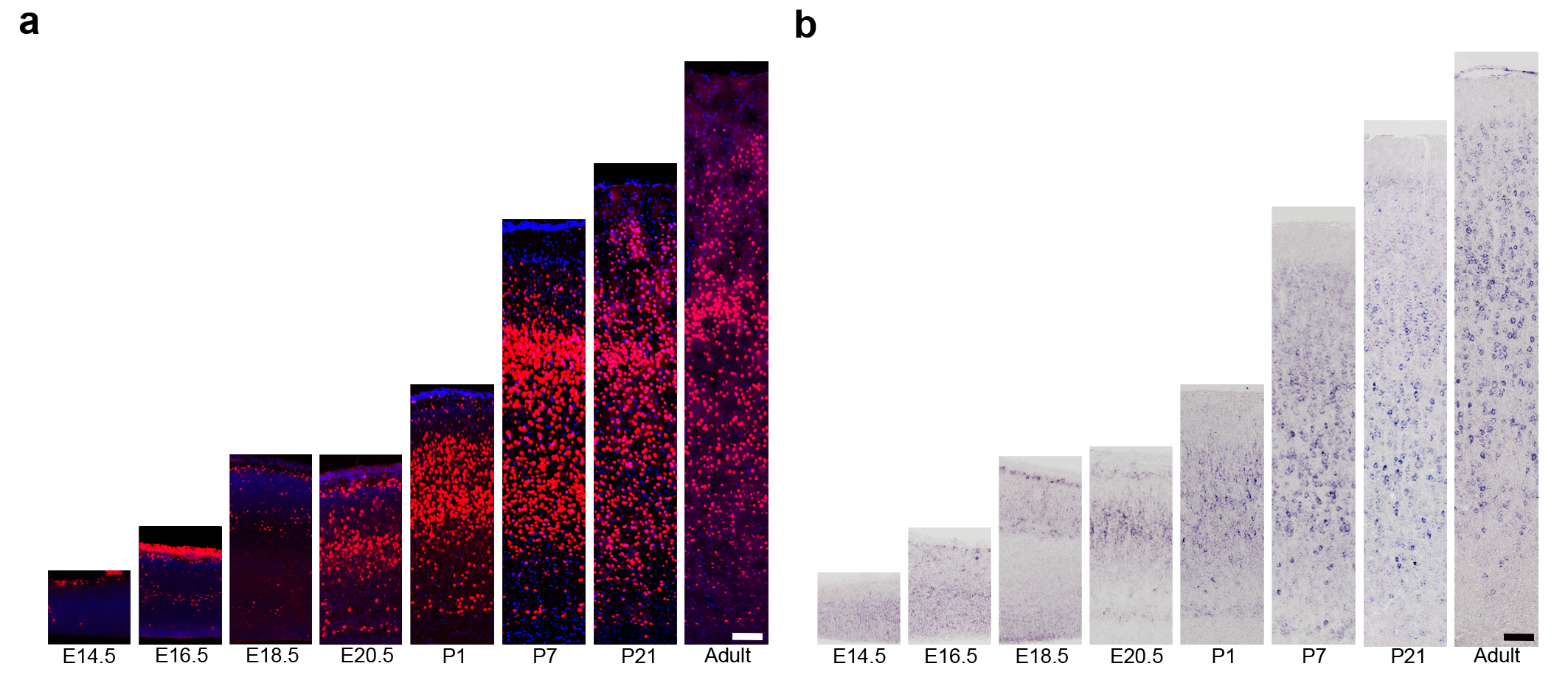

Supplement: Supplementary file 5 — Supplementary file: Fig. S1 [file 41401_2025_1588_MOESM5_ESM.jpg]

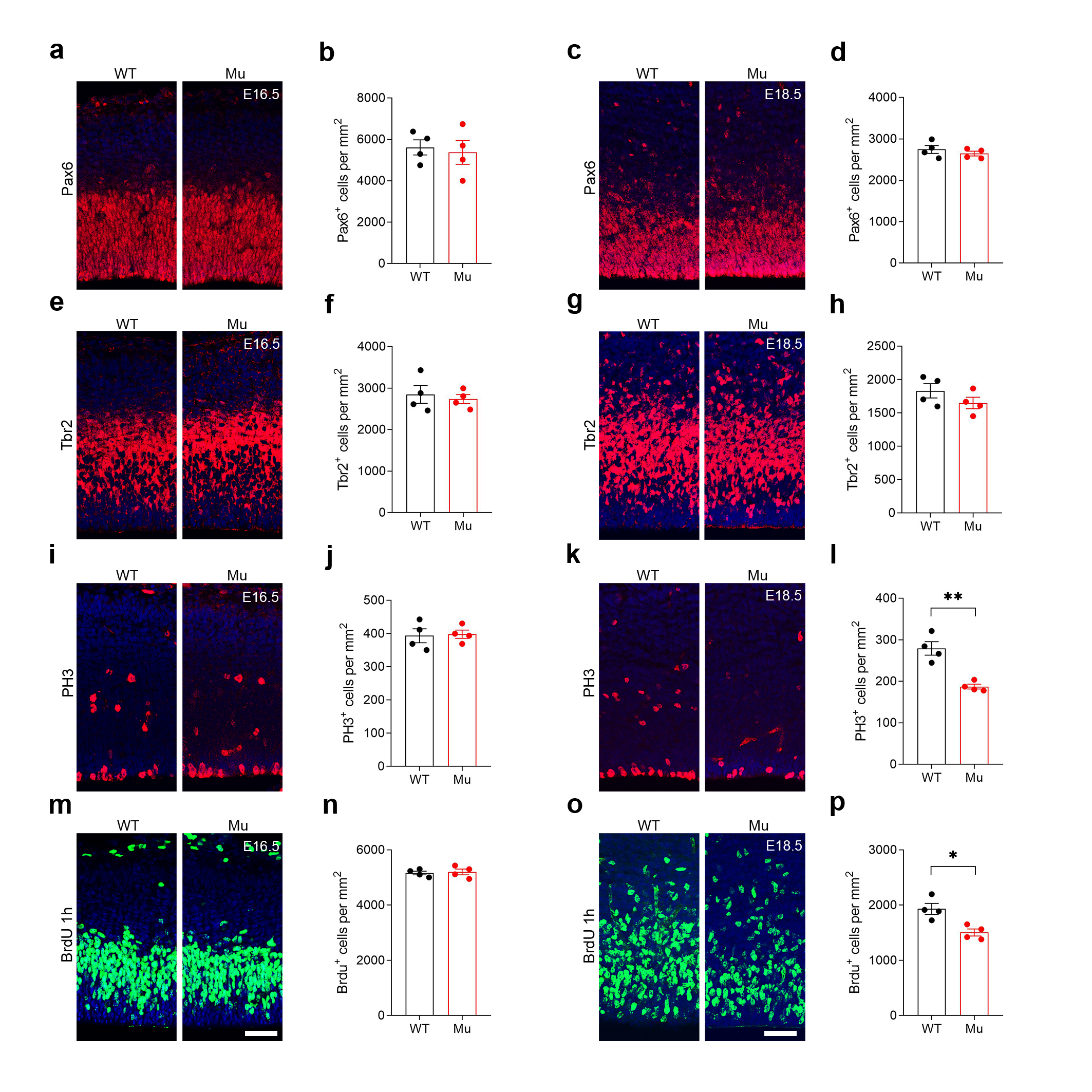

Supplement: Supplementary file 6 — Supplementary file: Fig. S2 [file 41401_2025_1588_MOESM6_ESM.jpg]

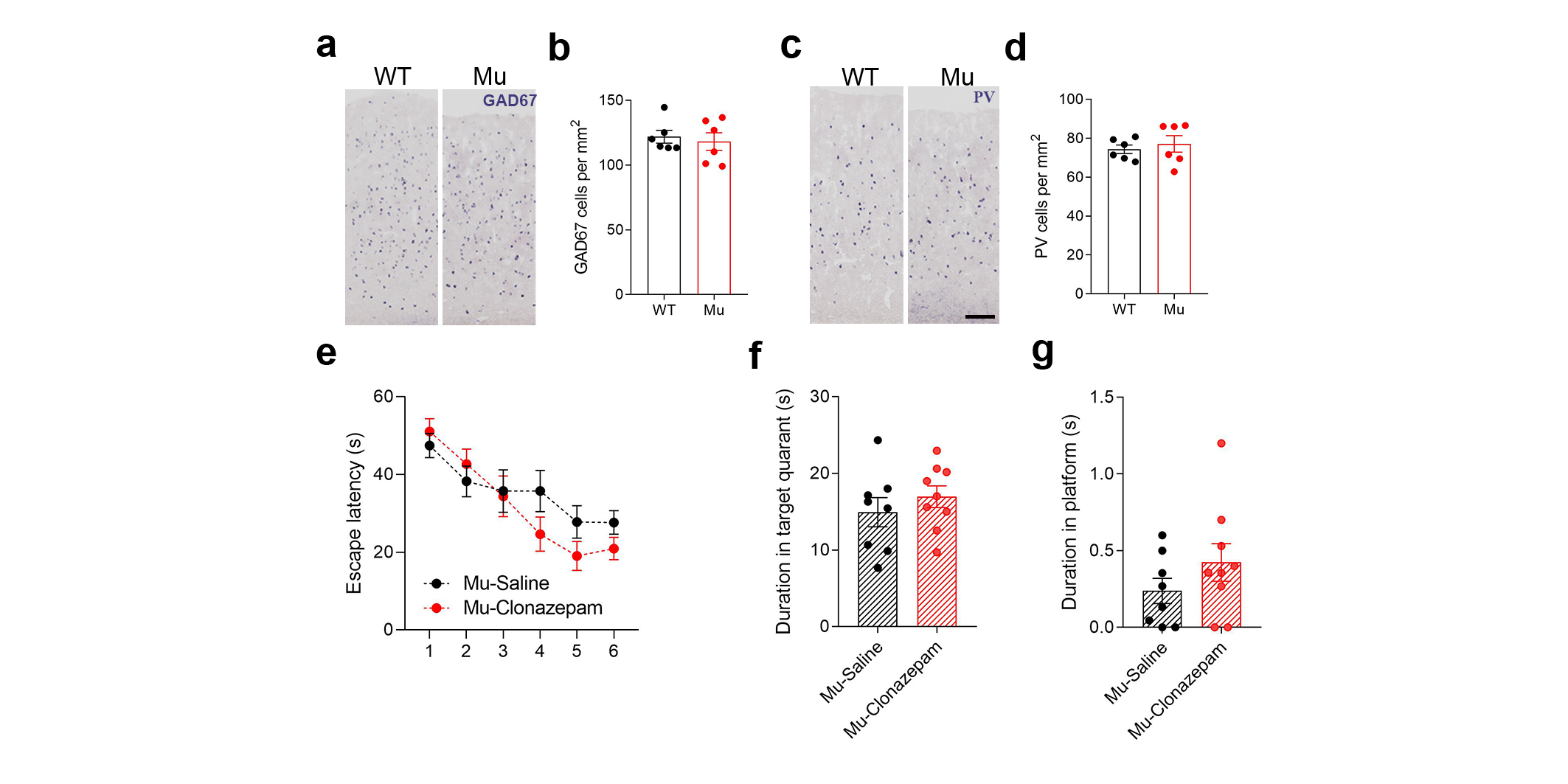

Supplement: Supplementary file 7 — Supplementary file: Fig. S3 [file 41401_2025_1588_MOESM7_ESM.jpg]
