# Supplementary material for: A rat Satb1 truncation causes neurodevelopmental abnormalities recapitulating the symptoms of patients with SATB1 mutations
Source: Acta Pharmacol Sin. 2025 Jun 26;46(12):3163–76. doi: 10.1038/s41401-025-01588-6 (PMC12644705; doi:10.1038/s41401-025-01588-6)
Supplement: Supplementary file 8 — Supplementary file Tables and Figure Legends [file 41401_2025_1588_MOESM8_ESM.docx]

**SUPPLEMENTARY FILE**

**Fig. S1.** Expression of Satb1 in the rat cortex revealed by immunohistochemistry and *in situ* hybridization.

(a) Immunostaining for Satb1 in the rat cortex at different stages. (b) *In situ* hybridization of Satb1 in the rat cortex at different stages.

**Fig. S2.** Reduced progenitor pool in the cortex of *Satb1* mutant rats at E18.5.

(a–d) Immunostaining for Pax6 at E16.5 (a, b) and E18.5 (c, d). No difference was detected in the number of Pax6+ cells at either stage. (e–h) Immunostaining for Tbr2 at E16.5 (e, f) and E18.5 (g, h). No difference was detected in the number of Tbr2+ cells at either stage. (i–l) Immunostaining for PH3 at E16.5 (i, j) or E18.5 (k, l). A decreased number of PH3+ cells was found at E18.5 but not at E16.5 in mutant rats compared with WT controls. (m–p) Immunostaining for BrdU at E16.5 (m, n) or E18.5 (o, p). A decreased number of BrdU+ cells was found at E18.5 but not at E16.5 in mutant rats compared with WT controls. N = 4 rats in each group. Student’s t test. *P < 0.05.

**Fig. S3.** Unaltered GAD67^+^/PV^+^ interneuron densities in *Satb1* mutant rats and the absence of a clonazepam-induced improvement in Morris water maze performance.

(a-d) GAD67^+^/PV^+^ interneuron densities were not significantly different between *Satb1* mutant and wild-type rats. (e-g) Clonazepam administration did not elicit significant improvements in the performance of *Satb1* mutant rats during either the training phase or probe trial.

**Table S1.** PCR primers used for real-time PCR.

**Table S2.** Differentially expressed genes between WT and *Satb1* mutant rats.

**Table S3.** Gene Ontology analysis of DEGs.

**Table S4.** Differentially expressed genes that overlapped with ID, ASD, FMRP, and synaptosome genes.
